# Squamous cell carcinoma arising from anal fistula in an HIV-positive individual: a case report

**DOI:** 10.1093/jscr/rjae686

**Published:** 2024-11-05

**Authors:** Jie Yang, Liman Zhang, Qiang Wang, Lili Wang

**Affiliations:** Anorectal Department, Shijiazhuang Traditional Chinese Medical Hospital, 233 Zhongshan Road, Shijiazhuang 050001, China; Anorectal Department, Shijiazhuang Traditional Chinese Medical Hospital, 233 Zhongshan Road, Shijiazhuang 050001, China; Anorectal Department, Shijiazhuang Traditional Chinese Medical Hospital, 233 Zhongshan Road, Shijiazhuang 050001, China; Anorectal Department, Shijiazhuang Traditional Chinese Medical Hospital, 233 Zhongshan Road, Shijiazhuang 050001, China

**Keywords:** fistula-associated squamous cell carcinoma, anal fistula, carcinogenesis, human immunodeficiency virus

## Abstract

Chronic anal fistula represents a prevalent form of perianal disease that frequently originates from perianal infection. Specifically, perianal abscesses that are inadequately or improperly treated are susceptible to the development of chronic anal fistulas. In HIV-infected individuals, an impaired immune system significantly diminishes the body’s capacity to combat infections and inflammation, thereby complicating the healing process of anal fistulas. Moreover, the impact of HIV on tissue repair results in a markedly prolonged healing process for wounds and tissue damage in these patients, exacerbating the difficulty in anal fistula resolution. Chronic anal fistulas that remain untreated for extended periods not only severely impair the patient’s quality of life but also pose an increased risk of malignant transformation.

## Introduction

Anal fistula is a common clinical condition; however, malignant transformation is rare. Chronic anal fistulas that persist for >10 years are considered a potential risk factor for fistula-associated cancers [1]. Several primary hypotheses have been proposed regarding the pathogenesis of malignancy in anal fistulas [2]: (i) abnormal hyperplasia of the congenital rectal mucosa, (ii) localized adenomatous hyperplasia of the anal glands, and (iii) carcinoma arising from rectal mucosal cells that have migrated to the anal fistula. A systematic search of the PubMed database from 1960 to 2024 revealed that most reports of anal fistula-related malignancies were mucinous adenocarcinomas, with squamous cell carcinoma being extremely rare. To date, there have only been three clinical reports of squamous cell carcinoma associated with anal fistulas, and no cases of squamous cell carcinoma arising from anal fistulas have been documented in HIV-positive patients. In this case report, we describe a patient with a chronic anal fistula and concurrent HIV infection, whose condition ultimately progressed to anal fistula-associated squamous cell carcinoma.

## Case report

**Figure 1:**
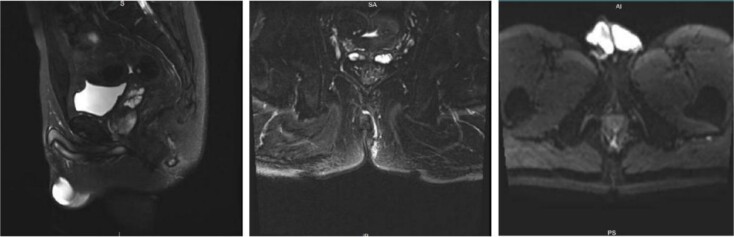
Preoperative perianal magnetic resonance imaging (MRI) examination.

**Figure 2:**
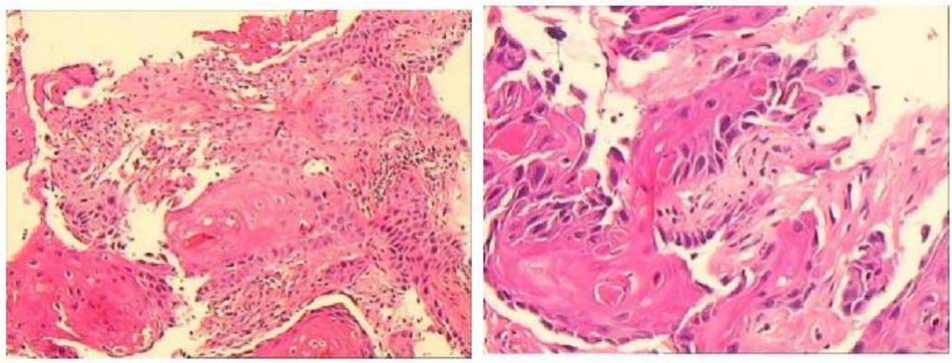
Postoperative pathology indicated squamous cell carcinoma.

A 43-year-old male patient was admitted in July 2022 due to an anal fistula. The patient had a 15-year history of recurrent perianal abscesses with prior surgical intervention in 2007, 2011, and 2012. However, following each surgery, the wounds failed to heal completely, resulting in persistent drainage of purulent fluid through the external orifices and intermittent recurrence of perianal abscesses. Two weeks before admission, the patient reported a significant increase in perianal pain. Notably, the patient had no history of inflammatory bowel disease, but had been diagnosed with HIV infection for 10 years. Physical examination revealed two external fistulous orifices located at the 3-o’clock and 9-o’clock positions, ~3 cm from the anal verge. Rectoscopy confirmed that the internal opening was situated within the anal crypt at the 6-o’clock position, and laboratory tests conducted upon admission did not reveal any abnormalities. Colonoscopy was performed and no underlying intestinal pathology was identified. Perianal magnetic resonance imaging revealed an intersphincteric fistula (Fig. 1).The patient underwent surgical intervention and the resected fistula tract was subjected to histopathological examination, which yielded a diagnosis of squamous cell carcinoma (Fig. 2). Consequently, the patient was not offered further surgical treatment at our institution but was referred to a specialized center for radiotherapy. During a 2-year follow-up after radiotherapy, the patient reported complete remission of the disease with no evidence of locally recurrent cancer cells. However, the surgical wound remained partially healed.

## Discussion

The relationship between cancer and anal fistulas can manifest in a number of ways [3]: (i) The presence of anal fistulas in conjunction with cancer in other regions of the colon does not necessarily indicate that the fistulas themselves are malignant. (ii) It is possible for cancer to manifest as an anal fistula, whereby a patient may initially be diagnosed with a fistula only to subsequently discover that it is in fact induced by cancer. (iii) It is possible for cancer to develop based on an existing fistula. Although the fistula itself may not initially be cancerous, a long-standing fistula could increase the risk of cancer development. In 1931, Rossier [4] first described six cases of chronic anal fistulas that had progressed to cancer, and proposed diagnostic principles for the development of cancer from anal fistulas. These principles were as follows: (i) persistent presence of the fistula, (ii) absence of tumors on the luminal surface of the rectal canal, and (iii) absence of malignant tissue in the internal opening of the fistula tract. In subsequent decades, further cases of fistula-derived cancer were documented, most commonly mucinous adenocarcinoma associated with anal fistulas. This is due to the fact that these fistulas are often linked with anal glands that have mucosecretory functions. Therefore, when an anal fistula persists and is subjected to chronic inflammatory stimuli, these glands may undergo malignant transformation or rectal mucosal cells may migrate via the anal glands into the fistula tract, forming mucinous adenocarcinoma [5, 6]. Reports of anal fistulas developing into squamous cell carcinoma are exceedingly rare, particularly those associated with HIV-infected infection.

In this case, the patient presented with a carcinoma at the site of an anal fistula. The patient had a history of chronic anal fistula for 15 years and a 10-year history of HIV infection. Preoperative colonoscopy ruled out the possibility of cancerous cells spreading from the anal canal and rectum to perianal tissue and gastrointestinal tract metastasis. Additionally, the examination eliminated the possibility that the cancer predated the fistula or that the anal fistula developed concurrently with anal canal carcinoma. Therefore, it is considered that the carcinoma arose secondary to chronic anal fistula.

The precise pathogenic mechanism underlying squamous cell carcinoma associated with anal fistulas is a vital factor in cancer development [7, 8]. It is widely acknowledged that chronic inflammation in an organ plays a role in the development and proliferation of cancer. It is widely accepted that persistent inflammation due to long-standing anal fistulas is a significant risk factor for malignant transformation of these structures [9]. As early as 1927, Brofeldt [10] put forth the hypothesis that any inflammatory stimulus in the perianal region could potentially lead to the development of leukoplakia and anal canal carcinoma. Infection of the anal glands is a prerequisite for malignant transformation of anal fistulas. Chronic inflammatory stimuli from persistent, recurrent episodes lead to tissue hyperplasia and fibrosis, resulting in atypical hyperplasia, which is the primary pathological basis for carcinogenesis of anal fistulas. If an anal fistula is not treated in a timely manner or is inadequately managed, it may result in the narrowing and twisting of the tract, impaired drainage, and false healing. This is accompanied by repeated infections and scar tissue proliferation, which further deteriorate the local blood supply. This results in the formation of a chronic suppurative infection site, which is characterized by repeated infections and significant fibrous tissue proliferation. This provides favorable conditions for the development of carcinogenesis. Long-term recurrent inflammatory stimuli result in metaplasia of the epithelial tissue of the infected fistula, which ultimately leads to tissue malignancy. Furthermore, this patient is also infected with HIV, which compromises the immune system, thereby reducing resistance to infections and inflammatory responses and slowing the wound and tissue repair process, which in turn exacerbates the persistence of inflammation [11].

The diagnosis of cancers associated with fistulas is challenging. Biopsy of the fistula tract is essential for diagnosis and early intervention [12]. In addition, all excised fistula tissues should undergo pathological examination, particularly those that have been present for a long time. Although there are few studies in the literature on squamous cell carcinoma associated with anal fistulas, treatment protocols for other fistula-associated cancers suggest that neoadjuvant/adjuvant radiochemotherapy and abdominoperineal resection remain the primary treatments [13]. Although the patient in this case did not receive further treatment in our department, the follow-up showed that he was cured by radiotherapy alone, with no tumor cells detected at the focal site, highlighting the therapeutic value of radiation.

This article presents the case of a patient with chronic anal fistula that ultimately progressed to fistula-associated squamous cell carcinoma, which suggests a potential link between HIV infection and the development of fistula-related cancer. The precise etiology and mechanisms underlying malignant transformation of anal fistulas remain unclear. Prognosis is dependent on early diagnosis and treatment, which emphasizes the importance of biopsy in the management of chronic anal fistulas.

## Data Availability

Data sharing is not applicable to this article, as no datasets were generated or analysed during the course of this study.
